# 2-((1H-Benzo[d]imidazol-2-yl)amino)benzo[d]thiazole-6-sulphonamides: a class of carbonic anhydrase II and VII-selective inhibitors

**DOI:** 10.1080/14756366.2023.2174981

**Published:** 2023-02-10

**Authors:** Morteza Abdoli, Claudiu T. Supuran, Raivis Žalubovskis

**Affiliations:** a Institute of Technology of Organic Chemistry, Faculty of Materials Science and Applied Chemistry, Riga Technical University, Riga, Latvia; b NEUROFARBA Department, Pharmaceutical and Nutraceutical Section, University of Florence, Florence, Italy; c Latvian Institute of Organic Synthesis, Riga, Latvia

**Keywords:** Carbonic anhydrase isozyme VII, neuropathic pain, sulphonamides, guanidines, inhibitors

## Abstract

A small library of substituted cyclic guanidine incorporated benzothiazole-6-sulphonamides was synthesized. All obtained compounds were investigated for their inhibitory activity against the key brain-associated human carbonic anhydrase isoform hCA VII (a promising target for the treatment of neuropathic pain) and three isoforms expressed in brain and other tissues, hCA I, II, and IV. Sulphaguanidine derivatives **9a–d** were inactive on the all investigated isoforms while the primary sulphonamide containing guanidines **6a–c** and **7a–c** were inactive towards hCA IV but displayed inhibiting properties on hCA I, II, and VII with K_Is _values in the low nanomolar to micromolar ranges. The results indicated that isoforms hCA II and VII were potently and selectively inhibited by these compounds, whereas the cytosolic hCA I was less sensitive to inhibition. The derivatives reported in this study might be useful for design of more potent and selective inhibitors of hCA II and VII.

## GRAPHICAL ABSTRACT

**Figure:**
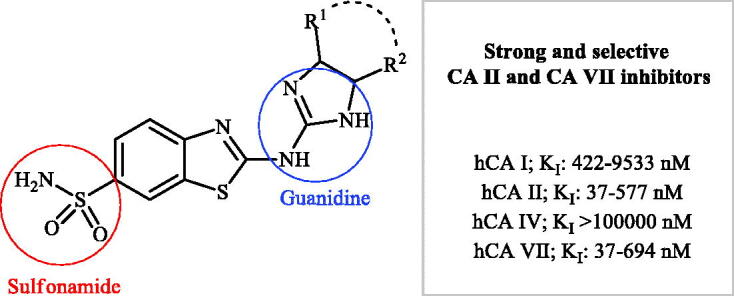

## Introduction

Human carbonic anhydrases (hCA, EC 4.2.1.1), are a large family of zinc-metalloenzymes with twelve catalytically active isoforms (hCA I, hCA II, hCA III, hCA IV, hCA VA, hCA VB, hCA VI, hCA VII, hCA IX, hCA XII, CA XIII, hCA XIV), which are vital for respiration, pH regulation, ion transport, metabolism, biosynthetic reactions and other physiological processes^1^. However, the abnormal activity of each isoform stimulates the initiation of various pathological processes in which a disturbed cellular pH buffering or metabolism plays a significant role^2^. Therefore, hCAs are regarded as promising therapeutic targets for many diseases, and the design of isoform-selective inhibitors has attracted the attention of researchers over the last 40 years^3^. In this context, brain-associated isoform CA VII, has been recently validated as an important target in neuropathic pain^4^, a disease which results from damage or dysfunction of the nervous system and whose patients suffer from the lack of effective treatment options^5^^,^^6^.

**Figure 1.**
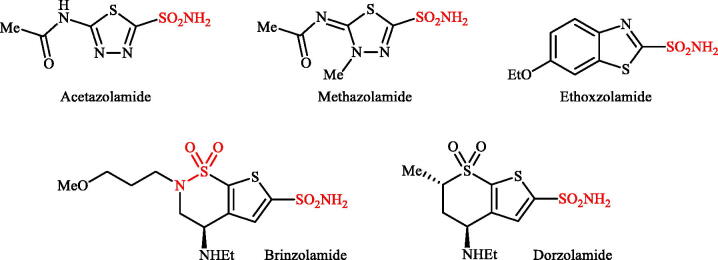
Chemical structures of acetazolamide, methazolamide, ethoxzolamide, brinzolamide, and dorzolamide.

**Figure 2.**
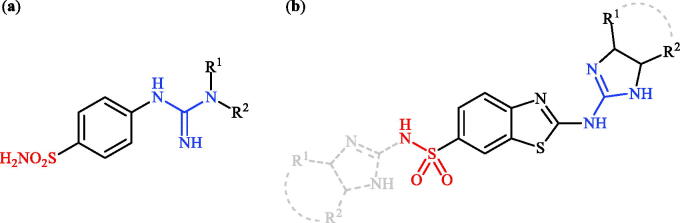
(a) General structure of 4-(3-alkyl/benzyl-guanidino)benzenesulphonamides developed by our group as hCA VII selective inhibitors; (b) general structure of cyclic guanidine incorporated benzothiazole-6-sulphonamides discussed in the paper.

Sulphonamides are the largest group of sulphur-containing drugs and the chief class of zinc-binding carbonic anhydrases inhibitors (CAIs)^7^^,^^8^. Acetazolamide, methazolamide, ethoxzolamide, brinzolamide, and dorzolamide were the earliest drugs approved as carbonic anhydrase inhibitors and still currently used in the treatment of various diseases such as glaucoma, duodenal ulcers, epilepsy and as diuretics^8^. As shown in Figure 1, the structural similarity of all five drugs is that they share the sulphonamide moiety bound to a heterocycle. High inhibitory potency against hCA VII is another similarity of these clinically used drugs^9^. However, they are not isoform selective inhibitors and are active against the majority of isoforms, with inhibition constants in low nanomolar range. Due to their poor selectivity against a single isoform, another thing they have in common is the multitude of side effects. Ethoxzolamide is the most potent hCA VII inhibitor in this series (K_I_ of 0.8 nM). However, it also inhibits hCA I, II, IV–VII, IX, and XII–XIV in moderate to low nanomolar ranges. Therefore, the search for structural improvement of this drug to produce less-toxic, more efficient, and isoform selective agents is an ongoing struggle^10^. Very recently, we disclosed that incorporation of the guanidine moieties as tails to classical benzenesulphonamide scaffolds improved selectivity against hCA VII versus ubiquitous hCA I and CA II (Figure 2(a))^11^. Considering all these facts and in connection with our works in the development of selective CAIs^12^, with the objectives of development of hCA VII selective inhibitors, in this study, we present the synthesis of three different sets of novel cyclic guanidine incorporated benzothiazole-6-sulphonamides (Figure 2(b)) and evaluate their capability to inhibit three cytosolic isozymes (hCA I, II, and VII) and one membrane-bound isozyme (hCA IV).

## Results and discussion

### Chemistry

The rationale for obtaining the novel compounds reported here consisted in replacing phenyl ring in the 4-guanidinobenzenesulphonamide scaffold, a structure recently reported by our group as selective inhibitor of hCA VII^11^, by a heteroaryl ring, leading thus to guanidine-heteroarene sulphonamide which presumably show enhanced hCAs inhibitory activity.

**Scheme 1.**
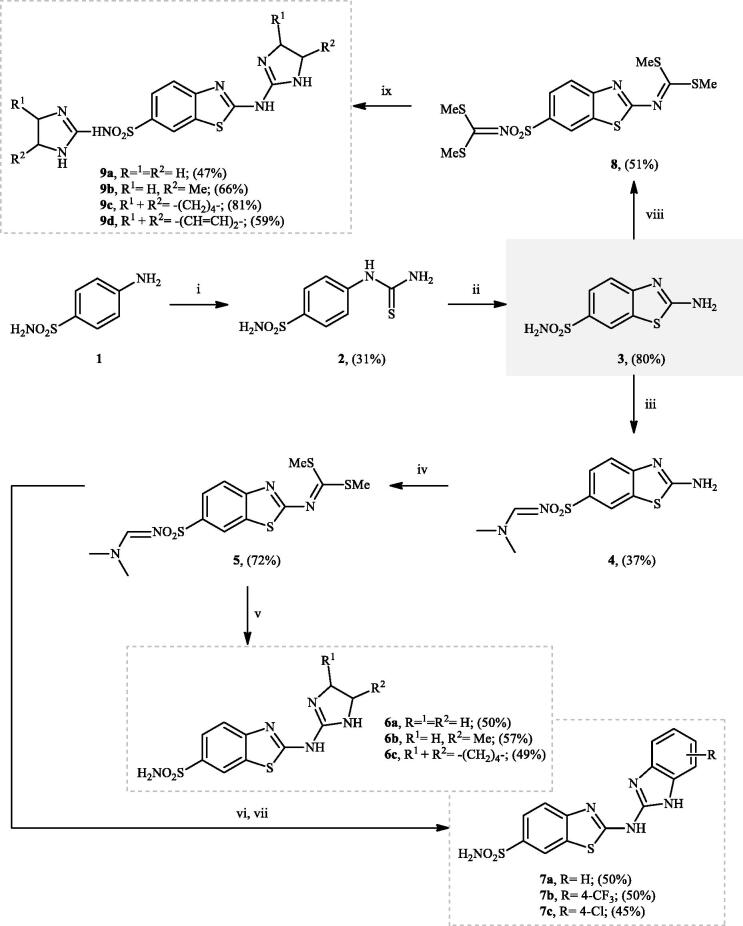
Reagents and conditions: (i) KSCN (1 equiv.), 3.5 M HCl, 90 °C, 3 h; (ii) Br_2_ (1.5 equiv.), CHCl_3_, reflux, 4.5 h; (iii) DMF-DMA (1 equiv.), DMF, 0 °C, 2.5 h; (iv) CS_2_ (1.7 equiv.), KOH (2.5 equiv.), MeI (2.5 equiv.), DMF/H_2_O (3:1); (v) aliphatic diamine (10 equiv.), DMSO, 120 °C, 15 h; (vi) 1,2-phenylenediamine (8 equiv.), DMSO, 120 °C, 20 h; (vii) N_2_H_4_.H_2_O, r.t., 1 h; (viii) CS_2_ (4 equiv.), KOH (5 equiv.), MeI (5 equiv.), DMF/H_2_O (3:1); (ix) aliphatic diamine (15 equiv.), DMSO, 120 °C, 15 h.

The synthesis of three different sets of 2-((imidazolyl)amino)benzothiazole-6-sulphonamides **6**, **7**, and **8** was performed as illustrated in Scheme 1. 4-Thioureidobenzenesulphonamide **2** was prepared by the reaction of 4-aminobenzenesulphonamide **1** with KSCN under acidic conditions. Consequently, the intermediate **2** was treated with Br_2_ using CHCl_3_ as a solvent to afford the key intermediate **3** which upon treatment with *N,N*-dimethylformamide *N,N*-dimethylacetamide (DMF-DMA) in DMF provided the *N*-protected sulphonamide intermediate **4**. Subsequently, the intermediate **4** was reacted with CS_2_ then MeI to yield dimethyl carbonimidodithioate **5**. The reaction between intermediate **5** and over-stoichiometric amounts of simple aliphatic diamines at elevated temperature was then performed to afford heteroayl sulphonamide-substituted cyclic guanidines **6** in moderate yields. Similarly, the intermediate **5** was reacted with various 1,2-phenylenediamines to give fluorescent 2-((benzoimidazol-2-yl)amino)benzothiazole-6-sulphonamides **7**. However, in these cases, an additional deprotection step was required. Finally, double *N*-dithiocarbonatation of 2-aminobenzothiazole-6-sulphonamide **3** with CS_2_/MeI was carried out to form intermediate **8** which was subsequently reacted with 1,2-diamines to afford the desired *N*-(imidazol-2-yl)-2-((imidazol-2-yl)amino)benzothiazole-6-sulphonamides **9**.

### Carbonic anhydrase inhibition

The inhibition data against four human CA isoforms expressed in brain, hCA I, II, IV (off-target) and VII (target for neuropathic pain drug discovery) with the newly synthesised sulphonamides **6a–c**, **7a–c**, and **9a–d** along with the reference drug acetazolamide (AAZ), are shown in Table 1.

**Table 1.** Inhibition data of human CA isoforms CAI, II, IV and VII with benzothiazole-6-sulphonamide substituted five-membered (bi)cyclic guanidines **6**, **7**, **9** using acetazolamide (AAZ) as standard drug.

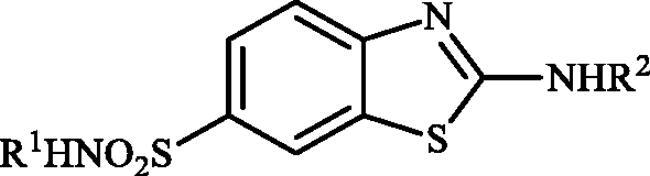						
Compound	R^1^	R^2^	K_I_ (nM)^a^			
			hCA I	hCA II	hCA IV	hCA VII
**6a**	–H	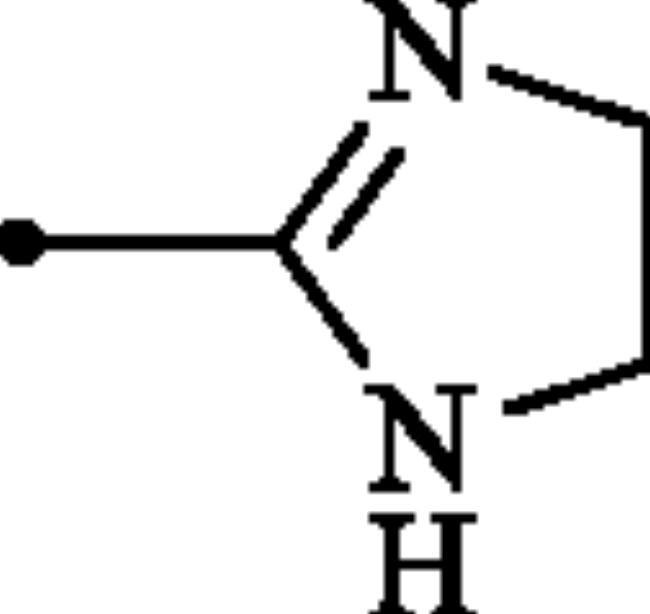	422.4	37.6	>100 000	56.3
**6b**	–H	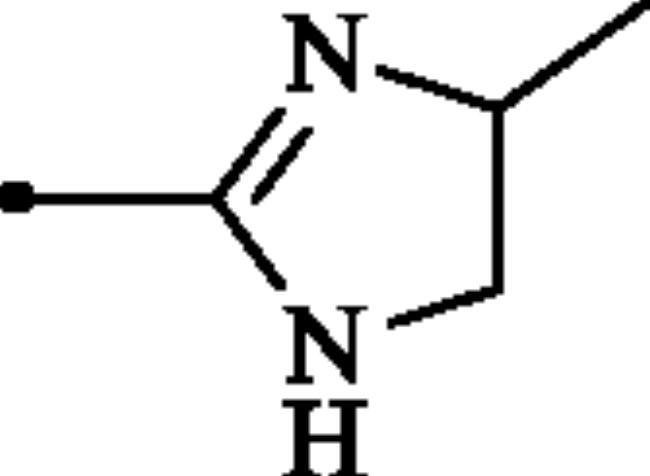	568.5	48.1	>100 000	37.4
**6c**	–H	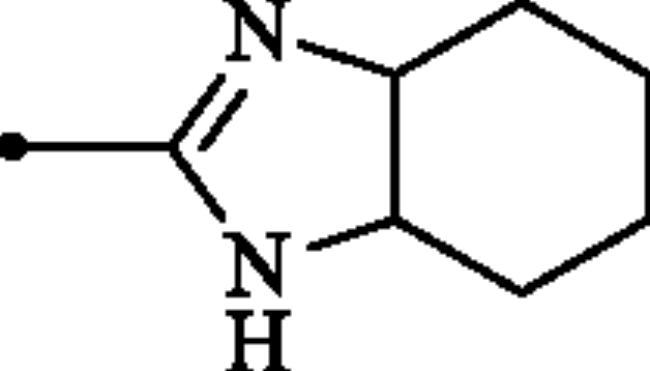	7590	65.6	>100 000	82.5
**7a**	–H	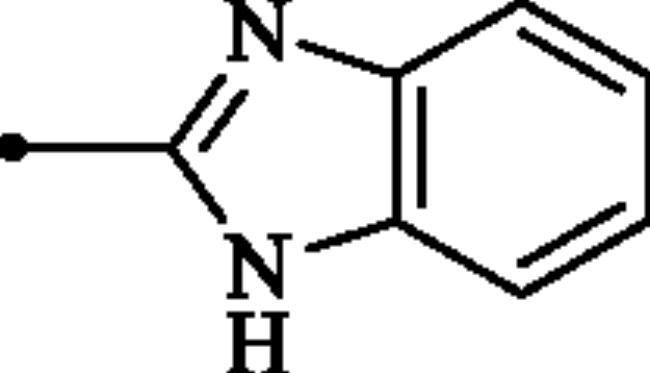	4927	84.0	>100 000	66.9
**7b**	–H	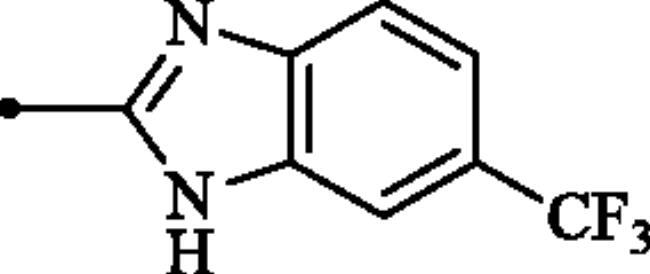	8102	375.0	>100 000	451.9
**7c**	–H	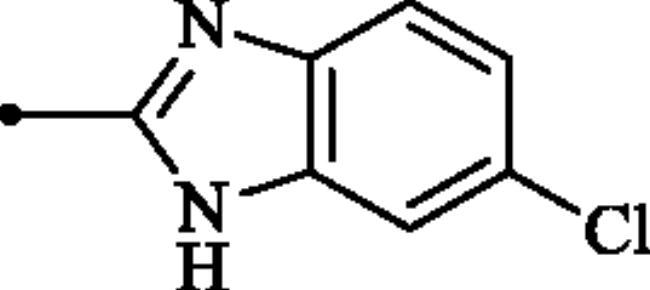	9533	577.6	>100 000	694.4
**9a**	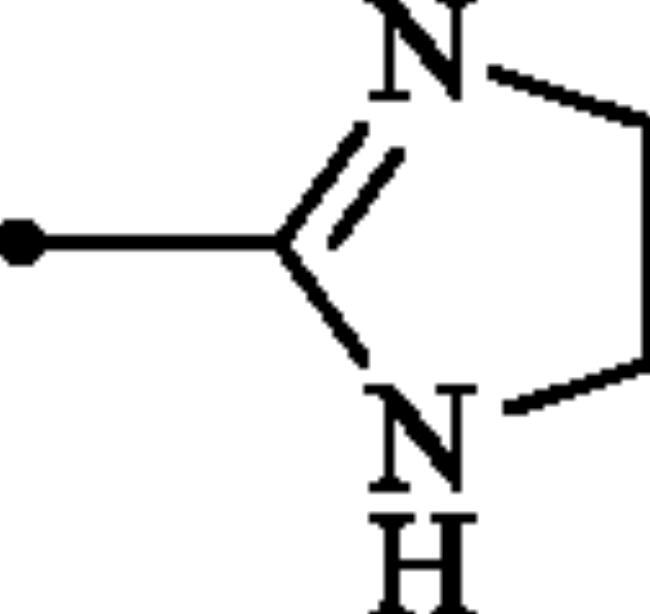	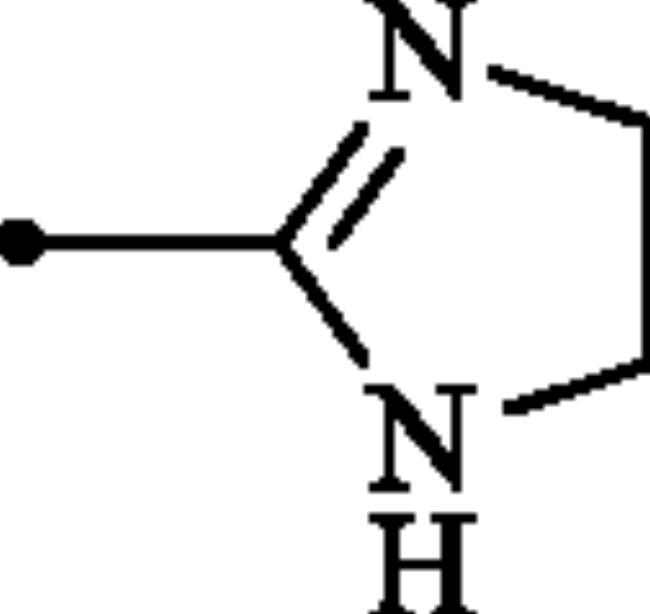	>100 000	>100 000	>100 000	>100 000
**9b**	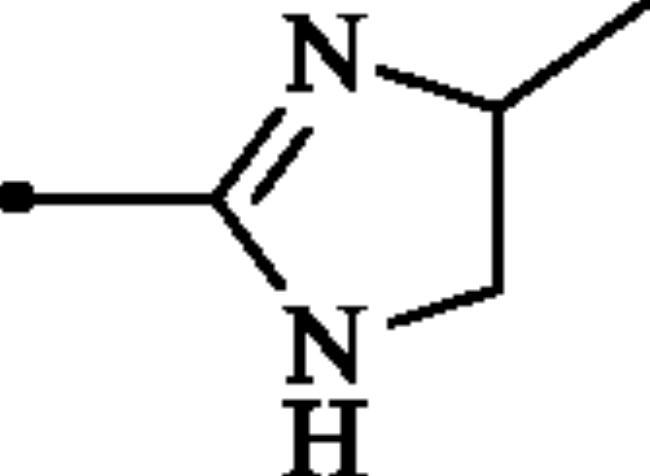	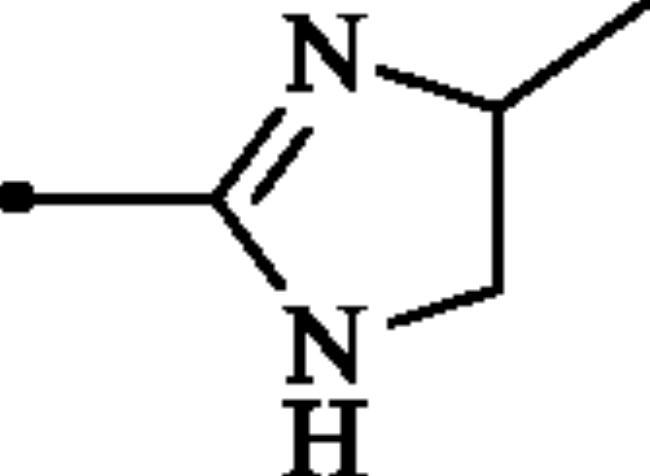	>100 000	>100 000	>100 000	>100 000
**9c**	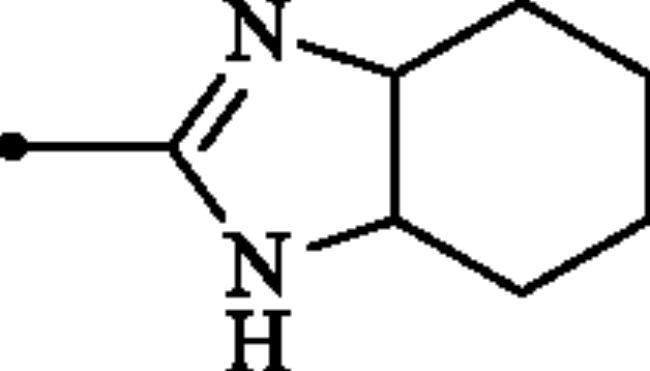	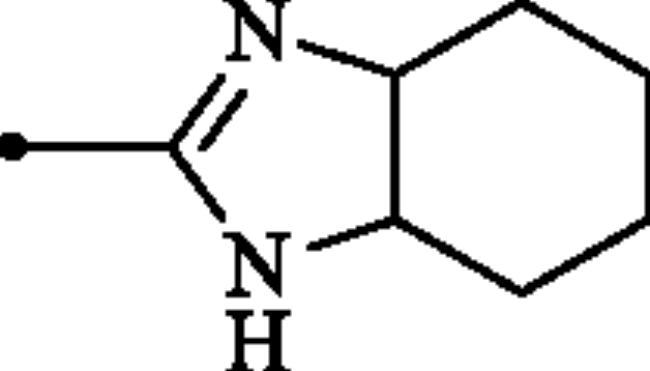	>100 000	>100 000	>100 000	>100 000
**9d**	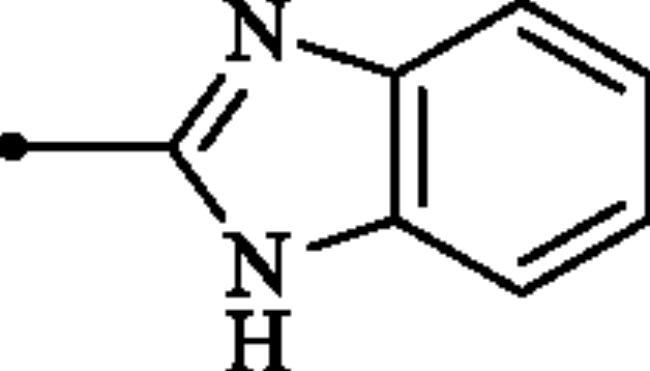	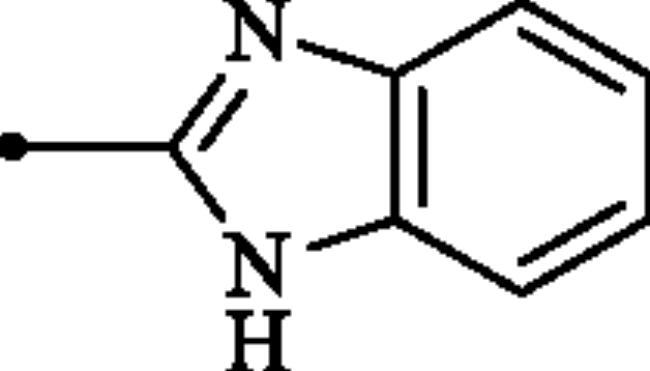	>100 000	>100 000	>100 000	>100 000
**AAZ**	–	–	250	12.5	74	2.5

^a^Mean from three different assays, by a stopped flow technique (errors were in the range of ± 5–10% of the reported values).

The following structure activity relationship (SAR) can be figured out from the inhibition data of Table 1:

- Among the entire series, only compounds bearing primary sulphonamide moiety **6a–c** and **7a–c** were moderately inhibited the cytosolic isoform hCA I, whereas sulphaguanidine derivatives **9a–d** were entirely inactive against this isoform (K_I_ > 100 µM). The SAR showed that compounds containing imidazoline core **6a–c** displayed better inhibitory capability against this isoform, with K_Is_ ranging between 442.4 and 7590 nM, as compared to the benzimidazoline-substituted ones **7a–c** (K_Is_ of 4927–9533 nM). Notably, the inhibitory potency was reduced significantly when any functionality was presented on the periphery of(benz)imidazoline rings. As shown in Table 1, 2-((4,5-dihydro-imidazol-2-yl)amino)benzothiazole-6-sulphonamide **6a** exhibited the best activity of all the evaluated compounds, albeit approximately two-fold less effective than standard drug, AAZ.
- The physiologically most relevant isoform hCA II was inhibited by compounds **6a–c** and **7a–c** more than hCA I (K_Is_ in the range 37.6–577.6 nM). The results showed that sulphaguanidines **9a–d** were also inactive against this isoform. Again imidazoline-incorporated sulphonamides **6a–c** demonstrated superior inhibitory activities (K_Is_ in the range 37.6–65.6 nM) compared to benzimidazoline-substituted derivatives **7a–c** (K_Is_ of 84.0–577.6 nM). Once more, the optimal substitution seems to be an unsubstituted imidazoline ring, in **6a**, which is the most effective hCA II inhibitor in the series investigated here.
- As seen from data of Table 1, none of the tested compounds showed inhibitory action against the transmembrane isoform hCA IV (K_I_ > 100 µM), which is considered as being an off-target isoform in our study. Notably, AAZ shows low nanomolar inhibitory activity against this isoform with a K_I_ of 74nM.
- Similar to the inhibitory pattern of investigated compounds against ubiquitous CA I and CA II, sulphaguanidine derivatives **9a–d** failed to inhibit the neuropathic pain associated hCA VII. However, the rest of the derivatives **6a–c** and **7a–c** effectively inhibited this isoform, with K_Is_ values ranging from 37.4to 694.4 nM. Although again analogues **6a–c** exhibited better inhibitory activity when compared to compounds **7a–c**, in this case, the SAR was not very flat. Indeed, compound **6a** has the highest inhibitory effect for the off-target isoforms hCA I and hCA II while methyl-substituted compound **6b** showed superior inhibitory potency towards hCA VII. Although this compound displayed 15-fold higher selectivity for hCA VII over hCA I, it did not show significant selectivity towards hCA VII versus hCA II (hCA VII/hCA II ≈ 1.3).

## Conclusion

We have synthesised three small sets of novel cyclic guanidine incorporated benzothiazole-6-sulphonamides (**6a–c**, **7a–c**, and **9a–d**) and screened them against four human CA isoforms expressed in brain, hCA I, II, IV and VII. Among them, compounds bearing primary sulphonamide moiety **6a–c** and **7a–c** showed potent inhibitory activity against three cytosolic isoforms hCA I, II and VII whereas they did not display any inhibitory activity towards membrane-bound isoform hCA IV. The SAR indicated that generally compounds of series **6a–c** displayed better hCA inhibitory activity compared to the derivatives **7a–c**. It was also found that hCA II and VII were the considerably most sensitive to these inhibitors than hCA I. However, the newly developed compounds did not display remarkable selectivity towards hCA VII versus hCA II. Therefore, further optimisation and exploration of this kind of novel scaffolds required to development of new isoform-selective CAIs with better inhibition potency and fewer side effects.

## Experimental section

### Chemistry methods

Starting materials, reagents and solvents were purchased from commercial sources and used as received without any type of further purification. Thin-layer chromatography (TLC) was performed on silica gel, spots were visualised with UV light (254 and 365 nm). NMR spectra were recorded on Bruker 300 and 500 spectrometers in DMSO or DMF with chemical shifts values (*δ*) in ppm relative to tetramethylsilane (TMS). High-resolution mass spectra (HRMS) were recorded on a mass spectrometer with a Q-TOF micro mass analyser using the ESI technique.

### Synthesis

#### 4-Thioureidobenzenesulphonamide (2)


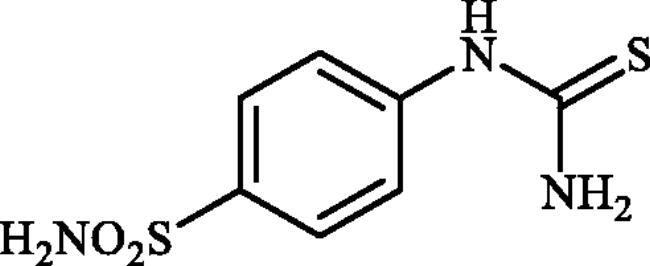
 4-Aminobenzensulphonamide (**1**) (30 g, 174.3 mmol) was dissolved in 3.5 M HCl (180 ml) under gentle warming. The solution was cooled down to r.t. and then KSCN (16.94 g, 174.3 mmol) was added to the reaction mixture and the mixture was refluxed for 3 h. After cooling to room temperature, the reaction mixture was diluted with ice-cold water (approximately 200 ml). The formed solids were collected by filtration, washed with water and air dried to afford **2** (12.1 g, 31%) as white powder.

^1^H NMR (300 MHz, DMSO-d_6_) *δ* = 7.32 (s, 2H), 7.69 (d, 2H, *J =* 8.6 Hz), 7.77 (d, 2H, *J =* 8.6 Hz), 10.02 (s, 1H) ppm ^13^C NMR (75 MHz, DMSO-d_6_) *δ* = 122.8, 127.3, 139.8, 143.9, 182.8 ppm MS (ESI) [M + H]^+^: m/z 232.0

#### 2-Aminobenzo[d]thiazole-6-sulphonamide (3)


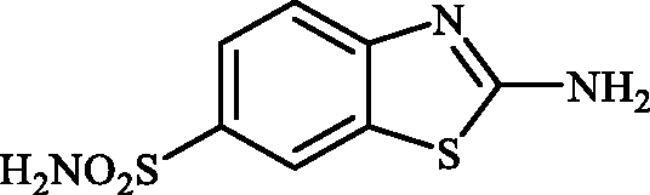
 To a suspension of 4-thioureidobenzenesulphonamide (**2**) (10 g, 43.3 mmol) in CHCl_3_ (120 ml) was added dropwise a solution of Br_2_ (3.27 ml, 64.9 mmol) in CHCl_3_ (15 ml) and stirred at 70 °C for 4.5 h. After cooling to rt volatiles were removed under reduced pressure to give a lumpy solid that was dissolved in H_2_O (120 ml). The aqueous solution was treated with ammonium hydroxide until the pH adjusted to greater than 9–10 and then stirred at 90 °C for 1 h. The formed precipitate was collected by filtration, washed with H_2_O and cold ethanol and dried under vacuum to afford compound **3** (7.9 g, 80%) as off-white solid.

^1^H NMR (300 MHz, DMSO-d_6_) δ = 7.25 (s, 2H), 7.47 (d, 1H, *J* = 8.4 Hz), 7.72 (d, 1H, *J* = 8.4 Hz), 7.90 (s, 2H), 8.17 (s, 1H) ppm ^13^C NMR (75 MHz, DMSO-d_6_) δ = 118.2, 120.1, 124.6, 131.9, 137.1, 156.4, 170.3 ppm HRMS (ESI) [M + H]^+^: *m*/*z* calcd for (C_7_H_8_N_3_O_2_S_2_) 230.0058. Found 230.0062.

#### N'-((2-aminobenzo[d]thiazol-6-yl)sulphonyl)-N,N-dimethylformimidamide (4)


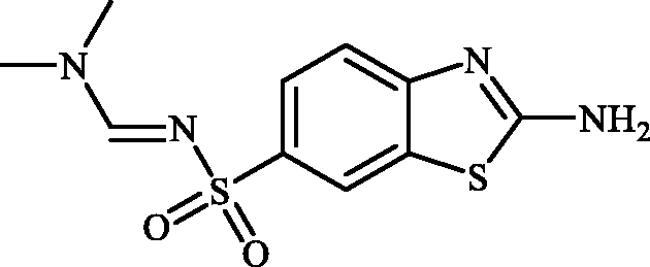
 To an ice-cooled stirred solution of 2-aminobenzo[d]thiazole-6-sulphonamide (**3**) (20 g, 87.3 mmol) in DMF (160 ml), DMF-DMA (11.6 ml, 87.3 mmol) was added dropwise and stirring continued at 0 °C for 2 h. After completion of the reaction, the mixture was extracted with DCM (3 × 100 ml). The combined organic layers were washed with water (1 × 100 ml) and concentrated under reduced pressure to 30 ml and then treated with CHCl_3_ (250 ml). The precipitated solid was collected by filtration, washed with CHCl_3_ (3 × 25 ml) and dried to afford the target product **4** (9.3 g, 37%) as yellowish powder.

Note, due to the precipitation of considerable amounts of the desired product by adding Na_2_SO_4_ to the organic extracts, extracts collected were not dried over Na_2_SO_4_ and directly concentrated under vacuum.

^1^H NMR (500 MHz, DMSO-d_6_) δ = 2.92 (s, 3H), 3.16 (s, 3H), 7.41 (d, 1H, *J* = 8.4 Hz), 7.62 (d, 1H, *J* = 8.4 Hz), 7.88 (s, 2H), 8.14 (s, 1H), 8.22 (s, 1H) ppm ^13^C NMR (125 MHz, DMSO-d_6_) δ = 35.9, 41.8, 118.0, 120.4, 124.8, 132.0, 135.8, 156.3, 160.4, 170.3 ppm HRMS (ESI) [M + H]^+^: *m*/*z* calcd for (C_10_H_13_N_4_O_2_S_2_) 285.0480. Found 285.0490.

#### Dimethyl (6-(N-((dimethylamino)methylene)sulphamoyl)benzo[d]thiazol-2-yl)carbonimidodithioate (5)


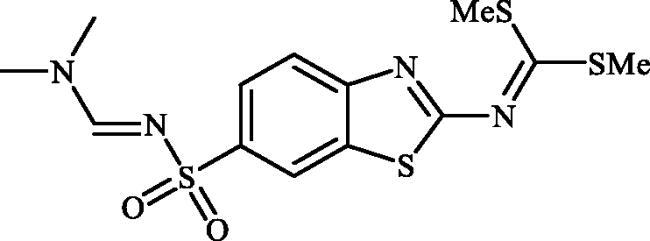
 To an ice cooled solution of *N'*-((2-aminobenzo[*d*]thiazol-6-yl)sulphonyl)-*N,N*-dimethylformimidamide (**4**) (10 g, 35.2 mmol) and CS_2_ (3.6 ml, 59.8 mmol) in DMF (75 ml) was dropwise added a solution of KOH (4.75 g, 88.0 mmol) in H_2_O (25 ml) at a such rate that the temperature kept below 10 °C. After 1 h stirring, MeI (5.47 ml, 88.0 mmol) was dropwise added and the reaction mixture was stirred at the indicated temperature for another 1 h. Then water (60 ml) and hexanes (12 ml) were added to the mixture and the solution was vigorously stirred for 20 min. Subsequently, the solids formed in the organic phase were collected by filtration and washed with water and cold ethanol to afford compound **5** (9.85 g, 72%) as yellow solids.

^1^H NMR (500 MHz, DMSO-d_6_) δ = 2.67 (s, 6H), 2.95 (s, 3H), 3.19 (s, 3H), 7.84 (d, 1H, *J* = 8.4 Hz), 7.94 (d, 1H, *J* = 8.4 Hz), 8.28 (s, 1H), 8.50 (s, 1H) ppm ^13^C NMR (125 MHz, DMSO-d_6_) δ = 16.6, 36.0, 41.9, 121.5, 122.8, 125.0, 135.0, 139.6, 153.9, 160.7, 170.8, 178.4 ppm HRMS (ESI) [M + H]^+^: *m*/*z* calcd for (C_13_H_17_N_4_O_2_S_4_) 389.0234. Found 389.0247.

#### 2-((4,5-Dihydro-1H-imidazol-2-yl)amino)benzo[d]thiazole-6-sulphonamide (6a)


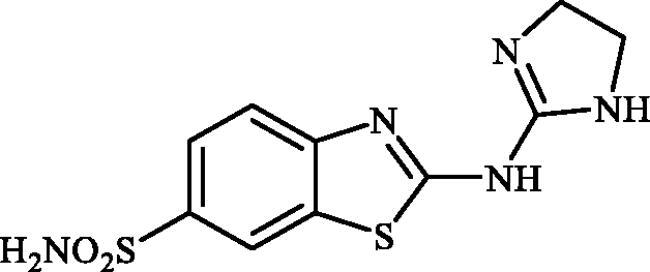
 A mixture of dimethyl (6-(*N*-((dimethylamino)methylene)sulphamoyl)benzo[*d*]thiazol-2-yl)carbonimidodithioate (**5**) (0.4 g, 1.03 mmol) and ethane-1,2-diamine (0.69 ml, 10.3 mmol) in DMSO (5.0 ml) was stirred at 120 °C for 15 h. After completion of the reaction, the mixture was cooled to room temperature and treated with water (25 ml) and then extracted with EtOAc (3 × 25 ml). The combined organic extracts were washed with water (3 × 25 ml) and concentrated to obtain a residue that was washed with Et_2_O to afford the compound **6a** (0.153 g, 50%) as white powder.

^1^H NMR (500 MHz, DMSO-d_6_) δ = 3.63 (s, 4H), 7.27 (s, 2H), 7.60 (d, 1H, *J* = 8.4 Hz), 7.74 (d, 1H, *J* = 8.4 Hz), 8.07 (s, 2H), 8.17 (s, 1H) ppm ^13^C NMR (125 MHz, DMSO-d_6_) δ = 42.7, 119.0, 119.9, 124.2, 132.0, 137.9, 155.5, 162.3, 177.1 ppm HRMS (ESI) [M + H]^+^: *m*/*z* calcd for (C_10_H_12_N_5_O_2_S_2_) 298.0432. Found 298.0439.

#### 2-((5-Methyl-4,5-dihydro-1H-imidazol-2-yl)amino)benzo[d]thiazole-6-sulphonamide (6b)


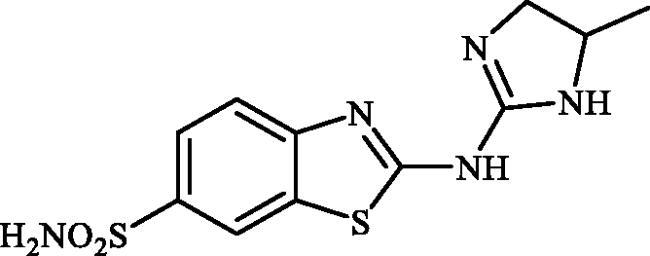
 A mixture of dimethyl (6-(*N*-((dimethylamino)methylene)sulphamoyl)benzo[*d*]thiazol-2-yl)carbonimidodithioate (**5**) (0.4 g, 1.03 mmol) and propane-1,2-diamine (0.88 ml, 10.3 mmol) in DMSO (5.0 ml) was stirred at 120 °C for 15 h. After completion of the reaction, the mixture was cooled to room temperature and treated with water (25 ml) and then extracted with EtOAc (3 × 25 ml). The combined organic extracts were washed with water (3 × 25 ml) and concentrated to obtain a residue that was washed with Et_2_O to afford compound **6b** (0.184 g, 57%) as white powder.

Note, due to the precipitation of considerable amounts of the desired product by adding Na_2_SO_4_ to the organic extracts, extracts collected were not dried over Na_2_SO_4_ and directly concentrated under vacuum.

^1^H NMR (500 MHz, DMSO-d_6_) δ = 1.27 (d, 3H, *J* = 5.6 Hz), 3.18–3.22 (m, 1H), 3.76 (d, 1H, *J* = 9.2 Hz), 4.03–4.08 (m, 1H), 7.27 (s, 2H), 7.60 (d, 1H, *J* = 8.4 Hz), 7.47 (d, 1H, *J* = 8.4 Hz), 8.07 (s, 1H), 8.14 (s, 1H), 8.17 (s, 1H) ppm ^13^C NMR (125 MHz, DMSO-d_6_) δ = 21.8, 49.8, 50.5, 118.9, 119.9, 124.2, 131.9, 137.9, 155.5, 161.2, 177.1 ppm HRMS (ESI) [M + H]^+^: *m*/*z* calcd for (C_11_H_14_N_5_O_2_S_2_) 312.0589. Found 312.0604.

#### 2-((3a,4,5,6,7,7a-hexahydro-1H-benzo[d]imidazol-2-yl)amino)benzo[d]thiazole-6-sulphonamide (6c)


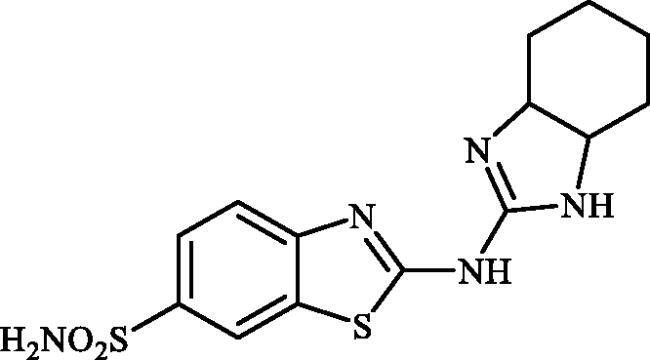
 A mixture of dimethyl (6-(*N*-((dimethylamino)methylene)sulphamoyl)benzo[*d*]thiazol-2-yl)carbonimidodithioate (**5**) (0.4 g, 1.03 mmol) and cyclohexane-1,2-diamine (1.24 ml, 10.3 mmol) in DMSO (5.0 ml) was stirred at 120 °C for 15 h. After completion of the reaction, the mixture was cooled to room temperature and treated with water (25 ml) and then extracted with EtOAc (3 × 25 ml). The combined organic extracts were washed with water (3 × 25 ml) and concentrated to obtain an orange solid that was dispersed in *t*-BuOMe (30 ml) and then filtered-off. The collected solution was treated with hexanes (40 ml) and the precipitated solids were collected by filtration and washed with hexanes and dried to afford compound **6c** (0.177 g, 49%) as white powder.

Note, due to the precipitation of considerable amounts of the desired product by adding Na_2_SO_4_ to the organic extracts, extracts collected were not dried over Na_2_SO_4_ and directly concentrated under vacuum.

^1^H NMR (500 MHz, DMSO-d_6_) δ = 1.28–1.52 (m, 4H), 1.73–1.84 (m, 2H), 2.07–2.16 (m, 2H), 3.12–3.21 (m, 2H), 7.28 (s, 2H), 7.66 (d, 1H, *J* = 7.7 Hz), 7.76 (d, 1H, *J* = 7.7 Hz), 8.20 (s, 1H), 8.22 (s, 2H) ppm ^13^C NMR (125 MHz, DMSO-d_6_) δ = 24.5, 29.8, 62.5, 119.3, 120.0, 124.3, 132.1, 138.2, 155.4, 163.3, 176.7 ppm HRMS (ESI) [M + H]^+^: *m*/*z* calcd for (C_14_H_18_N_5_O_2_S_2_) 352.0902. Found 352.0917.

#### 2-((1H-benzo[d]imidazol-2-yl)amino)benzo[d]thiazole-6-sulphonamide (7a)


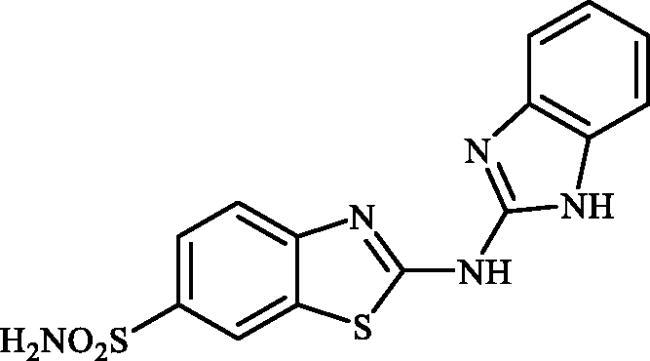
 A mixture of dimethyl (6-(*N*-((dimethylamino)methylene)sulphamoyl)benzo[*d*]thiazol-2-yl)carbonimidodithioate (**5**) (0.4 g, 1.03 mmol) and *o*-phenylenediamine (0.89 g, 8.24 mmol) in DMSO (5.0 ml) was stirred at 120 °C for 15 h. After completion of the reaction, the mixture was cooled to room temperature, hydrazine hydrate (2 ml) was dropwise added and the reaction mixture was stirred for 1 h. Then water (30 ml) and ethyl acetate (30 ml) were added to the mixture and the solution was stirred for 10 min and then remained still for a few minutes. Subsequently, the solids formed in the organic phase were collected by filtration and washed with EtOAc to afford compound **7a** (0.179 g, 50%) as white powder.

^1^H NMR (500 MHz, DMSO-d_6_) δ = 7.19–7.24 (m, 2H), 7.33 (s, 2H), 7.44–7.49 (m, 2H), 7.75 (d, 1H, *J* = 7.9 Hz), 7.83 (d, 1H, *J* = 7.9 Hz), 8.26 (s, 1H), 12.27 (s, 2H) ppm ^13^C NMR (125 MHz, DMF-d_7_) δ = 111.5, 118.2, 119.6, 122.6, 124.0, 131.0, 131.9, 137.8, 151.2, 154.8, 174.1 ppm HRMS (ESI) [M + H]^+^: *m*/*z* calcd for (C_14_H_12_N_5_O_2_S_2_) 346.0432. Found 346.0434.

#### 2-((6-(Trifluoromethyl)-1H-benzo[d]imidazol-2-yl)amino)benzo[d]thiazole-6-sulphonamide (7b)


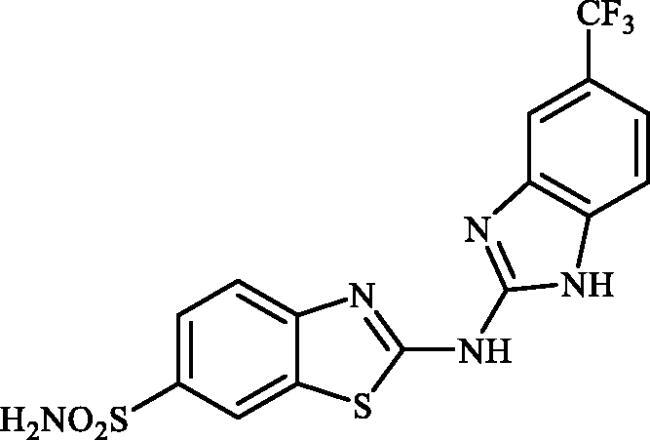
 A mixture of dimethyl (6-(*N*-((dimethylamino)methylene)sulphamoyl)benzo[*d*]thiazol-2-yl)carbonimidodithioate (**5**) (0.4 g, 1.03 mmol) and 4-(trifluoromethyl)benzene-1,2-diamine (1.45 g, 8.24 mmol) in DMSO (5.0 ml) was stirred at 120 °C for 15 h. After completion of the reaction, the mixture was cooled to room temperature, hydrazine hydrate (2 ml) was dropwise added and the reaction mixture was stirred for 1 h. Subsequently, the mixture was treated with water (50 ml) and the formed solids were collected by filtration, washed with *t*-BuOMe (50 ml) and dried under vacuum to afford compound **7b** (0.213 g, 50%) as pink powder.

^1^H NMR (500 MHz, DMSO-d_6_) δ = 7.37 (s, 2H), 7.51–7.87 (m, 5H), 8.34 (s, 1H), 12.48 (s, 2H) ppm ^13^C NMR (125 MHz, DMF-d_7_) δ = 109.4, 112.5, 117.8, 119.3, 120.0, 123.2 (q, *J* = 31.80 Hz), 124.3, 125.5 (q, *J* = 271.4 Hz), 131.4, 133.3, 135.9, 138.4, 152.6, 171.2 ppm HRMS (ESI) [M + H]^+^: *m*/*z* calcd for (C_15_H_11_N_5_O_2_F_3_S_2_) 414.0306. Found 414.0313.

#### 2-((6-Chloro-1H-benzo[d]imidazol-2-yl)amino)benzo[d]thiazole-6-sulphonamide (7c)


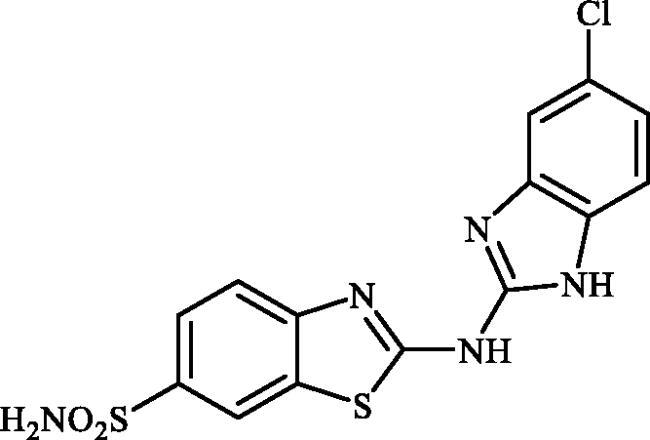
 A mixture of dimethyl (6-(*N*-((dimethylamino)methylene)sulphamoyl)benzo[*d*]thiazol-2-yl)carbonimidodithioate (**5**) (0.4 g, 1.03 mmol) and 4-chlorobenzene-1,2-diamine (1.17 g, 8.24 mmol) in DMSO (5.0 ml) was stirred at 120 °C for 15 h. After completion of the reaction, the mixture was cooled to room temperature, hydrazine hydrate (2 ml) was dropwise added and the reaction mixture was stirred for 1 h. Then water (30 ml) and ethyl acetate (30 ml) were added to the mixture and the solution was stirred for 10 min and then remained still for a few minutes. Subsequently, the solids formed in the organic phase were collected by filtration and washed with EtOAc to afford compound **7c** (0.177 g, 45%) as dark grey powder.

^1^H NMR (500 MHz, DMSO-d_6_) δ = 7.20 (d, 1H, *J* = 8.2 Hz), 7.44 (d, 1H, *J* = 8.2 Hz), 7.35 (s, 2H), 7.49 (s, 1H), 7.70 (d, 1H, *J* = 8.2 Hz), 7.85 (d, 1H, *J* = 8.2 Hz), 8.31 (s, 1H), 12.32 (s, 2H) ppm ^13^C NMR (125 MHz, DMF-d_7_) δ = 111.9, 113.0, 117.9, 119.8, 122.4, 124.2, 127.0, 131.6, 133.4, 138.2, 151.8, 153.3, 170.7, 172.2 ppm HRMS (ESI) [M + H]^+^: *m*/*z* calcd for (C_14_H_11_N_5_O_2_S_2_Cl) 380.0043. Found 380.0049.

#### Dimethyl (2-((bis(methylthio)methylene)amino)benzo[d]thiazol-6-yl)sulphonylcarbonimidodithioate (8)


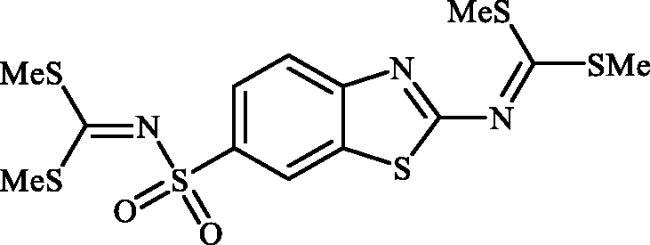
 To an ice cooled solution of 2-aminobenzo[*d*]thiazole-6-sulphonamide(**3**) (3 g, 1.31 mmol) and CS_2_ (3.16 ml, 5.24 mmol) in DMF (20 ml) was dropwise added a solution of KOH (3.67 g, 6.55 mmol) in H_2_O (10 ml). After 1 h stirring at 0 °C, MeI (4.08 ml, 6.55 mmol) was dropwise added and the reaction mixture was stirred at the indicated temperature for additional 1 h. Then the mixture was treated with water (100 ml) and the precipitated solid was collected by filtration under vacuum, washed with water, EtOH and then acetone to afford compound **8** (2.9 g, 37%) as yellowish powder.

^1^H NMR (300 MHz, 80 °C, DMSO-d_6_) δ = 2.61 (s, 3H), 2.62 (s, 3H), 2.68 (s, 3H), 2.69 (s, 3H), 7.95–8.0 (m, 2H), 8.61 (s, 1H) ppm ^13^C NMR (75 MHz, 80 °C, DMSO-d_6_) δ = 16.1, 16.6, 122.0, 122.4, 134.8, 136.7, 154.5, 170.9, 177.9, 186.0 ppm HRMS (ESI) [M + H]^+^: *m*/*z* calcd for (C_13_H_16_N_3_O_2_S_6_) 437.9567. Found 437.9574.

#### N-(4,5-Dihydro-1H-imidazol-2-yl)-2-((4,5-dihydro-1H-imidazol-2-yl)amino)benzo[d]thiazole-6-sulphonamide (9a)


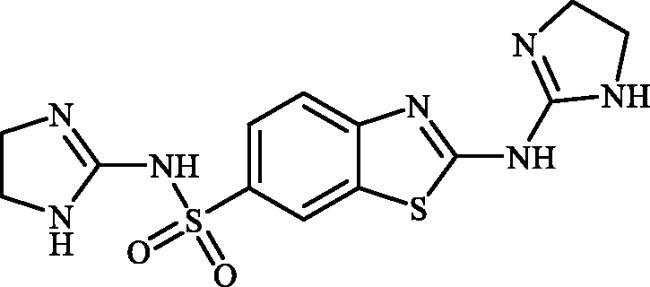
 A mixture of dimethyl (2-((bis(methylthio)methylene)amino)benzo[*d*]thiazol-6-yl)sulphonylcarbonimidodithioate (**8**) (0.4 g, 1.03 mmol) and ethane-1,2-diamine (0.916 ml, 15.45 mmol) in DMSO (5.0 ml) was stirred at 120 °C for 15 h. After completion of the reaction, the mixture was cooled to room temperature and treated with water (70 ml) and EtOAc (30 ml) and was vigorously stirred for 30 min. Subsequently, the solids formed in the organic phase were collected by filtration, washed with Et_2_O (30 ml) and dried under vacuum to compound **9a** (0.159 g, 47%) as pink powder.

^1^H NMR (500 MHz, DMSO-d_6_) δ = 3.46 (s, 4H), 3.62 (s, 4H), 7.48 (s, 2H), 7.54 (d, 1H, *J* = 7.8 Hz), 7.68 (d, 1H, *J* = 7.8 Hz), 8.04 (s, 2H), 8.13 (s, 1H) ppm ^13^C NMR (125 MHz, DMSO-d_6_) δ = 42.5, 42.7, 118.8, 119.9, 124.3, 131.7, 138.2, 155.1, 161.3, 162.2, 176.7 ppm HRMS (ESI) [M + H]^+^: *m*/*z* calcd for (C_13_H_16_N_7_O_2_S_2_) 366.0807. Found 366.0816.

#### N-(4,5-Dihydro-1H-imidazol-2-yl)-2-((4,5-dihydro-1H-imidazol-2-yl)amino)benzo[d]thiazole-6-sulphonamide (9b)


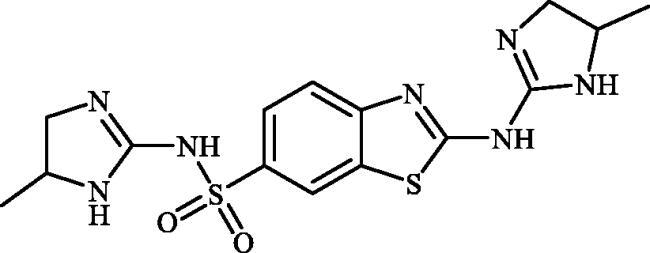
 A mixture of dimethyl (2-((bis(methylthio)methylene)amino)benzo[*d*]thiazol-6-yl)sulphonylcarbonimidodithioate (**8**) (0.4 g, 1.03 mmol) and propane-1,2-diamine (1.17 ml, 15.45 mmol) in DMSO (5.0 ml) was stirred at 120 °C for 15 h. After completion of the reaction, the mixture was cooled to room temperature and treated with water (70 ml) and EtOAc (30 ml) and was vigorously stirred for 30 min. Subsequently, the solids formed in the organic phase were collected by filtration, washed with Et_2_O (30 ml) and dried under vacuum to compound **9b** (0.240 g, 66%) as pink powder.

^1^H NMR (500 MHz, DMSO-d_6_) δ = 1.15 (d, 3H, *J* = 6.0 Hz), 1.27 (d, 3H, *J* = 6.0 Hz), 3.00–3.04 (m, 1H), 3.18–3.21 (m, 1H), 3.59 (t, 1H, *J* = 9.2 Hz), 3.76 (t, 1H, *J* = 9.2 Hz), 3.84–3.91 (m, 1H), 4.01–4.08 (m, 1H), 7.43 (s, 1H), 7.54 (d, 1H, *J* = 8.4 Hz), 7.63 (s, 1H), 7.68 (d, 1H, *J* = 8.4 Hz), 8.05 (s, 1H), 8.11 (s, 1H), 8.13 (s, 1H) ppm ^13^C NMR (125 MHz, DMSO-d_6_) δ = 21.6, 21.8, 49.5, 49.8, 50.4, 50.4, 118.8, 119.8, 124.2, 131.7, 138.3, 155.1, 160.2, 161.2, 176.7 ppm HRMS (ESI) [M + H]^+^: *m*/*z* calcd for (C_15_H_20_N_7_O_2_S_2_) 394.1120. Found 394.1133.

#### N-(3a,4,5,6,7,7a-hexahydro-1H-benzo[d]imidazol-2-yl)-2-((3a,4,5,6,7,7a-hexahydro-1H-benzo[d]imidazol-2-yl)amino)benzo[d]thiazole-6-sulphonamide (9c)


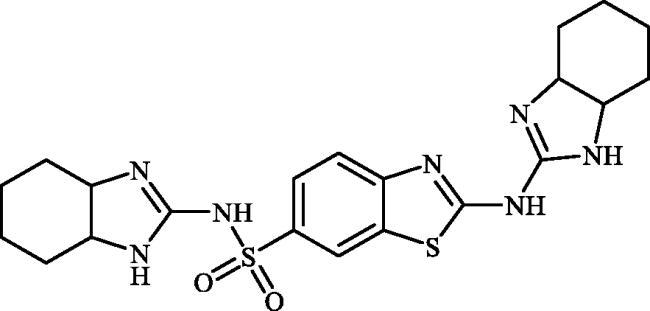
 A mixture of dimethyl (2-((bis(methylthio)methylene)amino)benzo[*d*]thiazol-6-yl)sulphonylcarbonimidodithioate (**8**) (0.4 g, 1.03 mmol) and cyclohexane-1,2-diamine (1.65 ml, 15.45 mmol) in DMSO (5.0 ml) was stirred at 120 °C for 15 h. After completion of the reaction, the mixture was cooled to room temperature and treated with water (100 ml) and the formed precipitate was collected by filtration, washed with H_2_O and *t*-BuOMe and dried under vacuum to afford compound **9c** (0.352 g, 81%) as white solid.

^1^H NMR (500 MHz, DMSO-d_6_) δ = 1.20–1.52 (m, 8H), 1.66–1.82 (m, 4H), 1.94–2.17 (m, 4H), 2.94–3.05 (m, 2H), 3.17–3.21 (m, 2H), 7.61 (s, 1H), 7.65–7.83 (m, 3H), 8.06–8.31 (m, 3H) ppm ^13^C NMR (125 MHz, DMSO-d_6_) δ = 24.4, 24.5, 29.6, 29.8, 62.3, 62.5, 119.2, 119.9, 124.3, 131.9, 138.3, 155.1, 162.6, 163.2, 176.4 ppm HRMS (ESI) [M + H]^+^: *m*/*z* calcd for (C_21_H_28_N_7_O_2_S_2_) 474.1746. Found 474.1750.

#### N-(3a,4,5,6,7,7a-hexahydro-1H-benzo[d]imidazol-2-yl)-2-((3a,4,5,6,7,7a-hexahydro-1H-benzo[d]imidazol-2-yl)amino)benzo[d]thiazole-6-sulphonamide (9d)


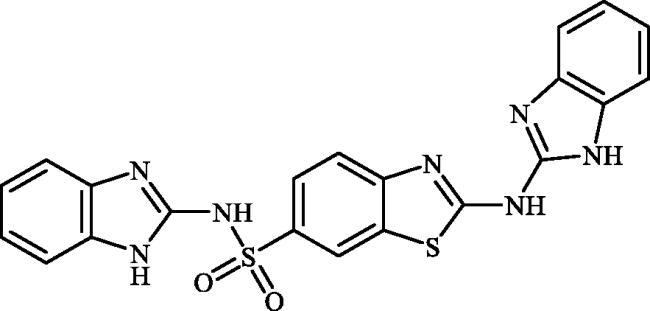
 A mixture of dimethyl (2-((bis(methylthio)methylene)amino)benzo[*d*]thiazol-6-yl)sulphonylcarbonimidodithioate (**8**) (0.4 g, 1.03 mmol) and *o*-phenylenediamine (1.48 g, 15.45 mmol) in DMSO (5.0 ml) was stirred at 120 °C for 15 h. After completion of the reaction, the mixture was cooled to room temperature, hydrazine hydrate (2 ml) was dropwise added and the reaction mixture was stirred for 1 h. Subsequently, the mixture was treated with water (100 ml) and the formed precipitate was collected by filtration, washed with H_2_O and *t*-BuOMe and dried under vacuum to afford compound **9d** (0.249 g, 51%) as grey solid.

^1^H NMR (500 MHz, DMSO-d_6_) δ = 6.94–7.60 (m, 8H), 7.63–7.98 (m, 2H), 8.36 (s, 1H), 11.95 (s, 2H), 12.23 (s, 2H) ppm ^13^C NMR (125 MHz, DMSO-d_6_) δ = 111.2, 111.6, 118.2, 119.4, 119.6, 122.8, 124.0, 130.4, 131.5, 131.9, 138.7, 151.3, 151.6, 154.3, 173.7 ppm HRMS (ESI) [M + H]^+^: *m*/*z* calcd for (C_21_H_16_N_7_O_2_S_2_) 462.0807. Found 462.0813.

### CA inhibition assays

An applied photophysics stopped-flow instrument has been used for assaying the CA-catalysed CO_2_ hydration activity^13^. Phenol red (at a concentration of 0.2 mM) was used as indicator, working at the absorbance maximum of 557 nm, with 20 mM Hepes (pH 7.5) as buffer and 20 mM Na_2_SO_4_ (for maintaining constant the ionic strength), following the initial rates of the CA-catalysed CO_2_ hydration reaction for a period of 10–100 s. The CO_2_ concentrations ranged from 1.7 to 17 mM for the determination of the kinetic parameters and inhibition constants. For each inhibitor, at least six traces of the initial 5–10% of the reaction have been used for determining the initial velocity. The uncatalysed rates were determined in the same manner and subtracted from the total observed rates. Stock solutions of inhibitor (0.1 mM) were prepared in distilled-deionised water, and dilutions up to 0.01 nM were done thereafter with the assay buffer. Inhibitor and enzyme solutions were preincubated together for 15 min at room temperature prior to assay in order to allow for the formation of the E-I complex. The inhibition constants were obtained by nonlinear least-squares methods using PRISM 3 and the Cheng–Prusoff equation, as reported earlier^14^ and represent the mean from at least three different determinations. All CA isoforms were recombinant ones obtained in-house as reported earlier^15^.

## References

[1] Aspatwar A, Tolvanen MEE, Barker H, Syrjänen L, Valanne S, Purmonen S, Waheed A, Sly WS, Parkkila S. Carbonic anhydrases in metazoan model organisms: molecules, mechanisms, and physiology. Physiol Rev. 2022;102(3):1327–1383. 35166161 10.1152/physrev.00018.2021

[2] (a) Supuran CT. Carbonic anhydrase inhibitors as emerging agents for the treatment and imaging of hypoxic tumors. Expert Opin Investig Drugs. 2018;27(12):963–970. (b) Supuran CT. Carbonic anhydrase inhibitors: an update on experimental agents for the treatment and imaging of hypoxic tumors. Expert Opin Investig Drugs. 2021;30(12):1197–1208. (c) Supuran CT. Carbonic anhydrases and metabolism. Metabolites. 2018;8(2):25. 10.1080/13543784.2018.1548608 30426805

[3] Winum JY. Targeting carbonic anhydrases. London (UK): Future Science Ltd; 2014. p. 18–33.

[4] Supuran CT. Carbonic anhydrase inhibition and the management of neuropathic pain. Expert Rev Neurother. 2016;16(8):961–968. 27211329 10.1080/14737175.2016.1193009

[5] Colloca L, Ludman T, Bouhassira D, Baron R, Dickenson AH, Yarnitsky D, Freeman R, Truini A, Attal N, Finnerup NB, et al. Neuropathic pain. Nat Rev Dis Primers. 2017;3:17002. 28205574 10.1038/nrdp.2017.2 PMC5371025

[6] Finnerup NB, Kuner R, Jensen TS. Neuropathic pain: from mechanisms to treatment. Physiol Rev. 2021;101(1):259–301. 32584191 10.1152/physrev.00045.2019

[7] Supuran CT. How many carbonic anhydrase inhibition mechanisms exist? J Enzyme Inhib Med Chem. 2016;31(3):345–360. 10.3109/14756366.2015.1122001 26619898

[8] Scott KA, Njardarson JT. Analysis of US FDA-approved drugs containing sulfur atoms. Top Curr Chem. 2018;376(1):5. 10.1007/s41061-018-0184-5 29356979

[9] Supuran CT. Carbonic anhydrases: novel therapeutic applications for inhibitors and activators. Nat Rev Drug Discov. 2008;7(2):168–181. 18167490 10.1038/nrd2467

[10] (a) Ibrahim DA, Lasheen DS, Zaky MY, Ibrahim AW, Vullo D, Ceruso M, Supuran CT, Abou El Ella DA. Design and synthesis of benzothiazole-6-sulfonamides acting as highly potent inhibitors of carbonic anhydrase isoforms I, II, IX and XII. Bioorg Med Chem. 2015;23(15):4989–4999. (b) Abdoli M, Angeli A, Bozdag M, Carta F, Kakanejadifard A, Saeidian H, Supuran CT. Synthesis and carbonic anhydrase I, II, VII, and IX inhibition studies with a series of benzo[d]thiazole-5- and 6-sulfonamides. J Enzyme Inhib Med Chem. 2017;32(1):1071–1078. (c) Manzoor S, Angeli A, Zara S, Carradori S, Rahman MA, Raza MK, Supuran CT, Hoda N. Development of benzene and benzothiazole-sulfonamide analogues as selective inhibitors of the tumor-associated carbonic anhydrase IX. Eur J Med Chem. 2022;243:114793.26048024

[11] Abdoli M, Giovannuzzi S, Supuran CT, Žalubovskis R. 4-(3-Alkyl/benzyl-guanidino)benzenesulfonamides as selective carbonic anhydrase VII inhibitors. J Enzyme Inhib Med Chem. 2022;37(1):1568–1576. 35635139 10.1080/14756366.2022.2080816 PMC9154774

[12] (a) Abdoli M, Bozdag M, Angeli A, Supuran CT. Benzamide-4-sulfonamides are effective human carbonic anhydrase I, II, VII, and IX inhibitors. Metabolites. 2018;8(2):37. 29857578 10.3390/metabo8020037 PMC6027465

[13] Khalifah RG. The carbon dioxide hydration activity of carbonic anhydrase. I. Stop-flow kinetic studies on the native human isoenzymes B and C. J Biol Chem. 1971;246(8):2561–2573. 4994926

[14] (a) Vermelho AB, da Silva Cardoso V, Ricci E, Junior Dos Santos EP, Supuran CT. Nanoemulsions of sulfonamide carbonic anhydrase inhibitors strongly inhibit the growth of *Trypanosoma cruzi*. J Enzyme Inhib Med Chem. 2018;33(1):139–146. (b) Nocentini A, Carta F, Tanc M, Selleri S, Supuran CT, Bazzicalupi C, Gratteri P. Deciphering the mechanism of human carbonic anhydrases inhibition with sulfocoumarins: Computational and experimental studies. Eur J Chem. 2018;24(31):7840–7844. (c) Angeli A, Carta F, Nocentini A, Winum JY, Zalubovskis R, Onnis V, Eldehna WM, Capasso C, Carradori S, Donald WA, Dedhar S, Supuran CT. Response to perspectives on the classical enzyme carbonic anhydrase and the search for inhibitors. Biophys J. 2021;120(1):178–181. (d) Nocentini A, Trallori E, Singh S, Lomelino CL, Bartolucci G, Di Cesare Mannelli L, Ghelardini C, McKenna R, Gratteri P, Supuran CT. 4-hydroxy-3-nitro-5-ureido-benzenesulfonamides selectively target the tumor-associated carbonic anhydrase isoforms IX and XII showing hypoxia-enhanced antiproliferative profiles. J Med Chem. 2018;61(23):10860–10874. (e) Chohan ZH, Munawar A, Supuran CT. Transition metal ion complexes of schiff-bases. Synthesis, characterization and antibacterial properties. Met Based Drugs. 2001;8(3):137–143. (f) Grandane A, Nocentini A, Werner T, Zalubovskis R, Supuran CT. Benzoxepinones: A new isoform-selective class of tumor associated carbonic anhydrase inhibitors. Bioorg Med Chem. 2020;28(11):115496.29192555

[15] (a) Ivanova J, Carta F, Vullo D, Leitans J, Kazaks A, Tars K, Žalubovskis R, Supuran CT. N-Substituted and ring opened saccharin derivatives selectively inhibit transmembrane, tumor-associated carbonic anhydrases IX and XII. Bioorg Med Chem. 2017;25(13):3583–3589. (b) Supuran CT. Carbon-versus sulphur-based zinc binding groups for carbonic anhydrase inhibitors? J Enzyme Inhib Med Chem. 2018;33(1):485–495. (c) Briganti F, Pierattelli R, Scozzafava A, Supuran CT. Carbonic anhydrase inhibitors. Part 37. Novel classes of isozyme I and II inhibitors and their mechanism of action. Kinetic and spectroscopic investigations on native and cobalt-substituted enzymes. Eur J Med Chem. 1996;31:1001–1010. (d) Pastorekova S, Casini A, Scozzafava A, Vullo D, Pastorek J, Supuran CT. Carbonic anhydrase inhibitors: the first selective, membrane-impermeant inhibitors targeting the tumor-associated isozyme IX. Bioorg Med Chem Lett. 2004;14(4):869–873. (e) Vullo D, Voipio J, Innocenti A, Rivera C, Ranki H, Scozzafava A, Kaila K, Supuran CT. Carbonic anhydrase inhibitors. Inhibition of the human cytosolic isozyme VII with aromatic and heterocyclic sulfonamides. Bioorg Med Chem Lett. 2005;15(4):971–976. (f) Gieling RG, Babur M, Mamnani L, Burrows N, Telfer BA, Carta F, Winum JY, Scozzafava A, Supuran CT, Williams KJ. Antimetastatic effect of sulfamate carbonic anhydrase IX inhibitors in breast carcinoma xenografts. J Med Chem. 2012;55(11):5591–5600. (g) Carta F, Supuran CT, Scozzafava A. Sulfonamides and their isosters as carbonic anhydrase inhibitors. Future Med Chem. 2014;6(10):1149–1165. (h) Sarikaya SB, Gülçin I, Supuran CT. Carbonic anhydrase inhibitors: Inhibition of human erythrocyte isozymes I and II with a series of phenolic acids. Chem Biol Drug Des. 2010;75(5):515–520. (i) Yıldırım A, Atmaca U, Keskin A, Topal M, Çelik M, Gülçin İ, Supuran CT. N-Acylsulfonamides strongly inhibit human carbonic anhydrase isoenzymes I and II. Bioorg Med Chem. 2015;23(10):2598–2605. (j) Innocenti A, Gülçin I, Scozzafava A, Supuran CT. Carbonic anhydrase inhibitors. Antioxidant polyphenols effectively inhibit mammalian isoforms I-XV. Bioorg Med Chem Lett. 2010;20(17):5050–5053. 28416101 10.1016/j.bmc.2017.04.007

